# Association of TIM-3 expression with glucose metabolism in Jurkat T cells

**DOI:** 10.1186/s12865-020-00377-6

**Published:** 2020-08-20

**Authors:** Mi Jin Lee, Su Jin Yun, Bokyoung Lee, Eun Jeong, Gyesoon Yoon, Kyongmin Kim, Sun Park

**Affiliations:** 1 Department of Microbiology, Ajou University School of Medicine, Suwon, 442-749 South Korea; 2 Department of Biomedical Sciences, The Graduate School, Ajou University, Suwon, 442-749 South Korea; 3 Department of Biochemistry & Molecular Biology, Ajou University School of Medicine, Suwon, 442-749 South Korea

**Keywords:** HAVCR2, Glycolysis, CD4^+^ T cell, Glutaminolysis, Glucose transporter

## Abstract

**Background:**

T cell activation is associated with increase in glycolysis and glutaminolysis. T cell immunoglobulin and mucin domain containing protein-3 (TIM-3), a T cell surface molecule, downregulates T cell activation and leads to insufficient immunity in cancer and chronic infection. TIM-3 regulates T cell activation possibly through alterations in metabolism; however, the relationship between TIM-3 expression and T cell metabolic changes has not been well studied.

**Results:**

We investigated the association between TIM-3 expression and metabolic changes by analyzing glucose metabolism, glutamine metabolism, and mitochondrial function in TIM-3 overexpressing or knockout Jurkat T cell lines relative to their control cell lines. Glucose uptake and consumption, and lactate release were downregulated by TIM-3 expression but upregulated by TIM-3 knockout. Concomitantly, the expression of the glucose transporter, Glut1, but not Glut2, 3, or 4 was altered by TIM-3 expression. However, TIM-3 expression alone could not account for the change in glutamine consumption, glutamate release, and mitochondrial mass, ROS production or membrane potential in these cell lines.

**Conclusion:**

Our results show the association of TIM-3 expression with T cell glucose metabolism. These results are significant in chronic infections and cancers where it is necessary to control TIM-3 expressing T cells.

## Background

T cell activation alters cellular metabolism [1]. Shifting of a T cell from a resting to an active state is accompanied by metabolic alterations to meet the increased demand for energy and macromolecule synthesis for cell proliferation and effector functions [2]. The metabolic alterations in activated T cells include increase in glucose and glutamine consumption [3, 4]. Glucose and glutamine are catabolized through glycolysis and glutaminolysis, respectively, to supply metabolic intermediates such as lactate and glutamate [3–5]. Glucose and glutamine are also directed to the hexosamine biosynthesis pathway to produce uridine diphosphate N-acetylglucosamine (UDP-GlcNAc), which is used for protein glycosylation. Blocking glucose and glutamine metabolism interferes with T cell activation [6, 7]. Downregulation of glycolysis inhibits the production of interferon- γ by activated CD4^+^ T cells as well as memory CD8^+^ T cells [8, 9]. Restriction of glucose and glutamine supply suppresses T cell proliferation through perturbation of protein glycosylation [6, 7]. Thus, T cell activation is closely associated with and regulated by metabolism.

Mitochondria play a critical role in T cell activation and differentiation [10]. Upon activation, the mitochondrial mass and DNA level increase in the T cell [10]. Antigenic stimulation leads to the relocation of mitochondria toward the immune synapse where they modulate calcium signaling and generate ATP for T cell activation [11, 12]. Mitochondria also produce reactive oxygen species (ROS) needed for the activation of transcription factors such as NFAT and AP-1 [13, 14]. Further, mitochondria are the organelles that provide the building blocks for macromolecule synthesis through the TCA cycle and oxidative phosphorylation [10]. Mitochondrial dysfunction impairs activation-induced T cell proliferation and differentiation [15].

TIM-3 is a transmembrane glycoprotein encoded by the HAVCR2 gene and it regulates T cell activation [16]. TIM-3 molecules were initially identified on terminally differentiated TH1 cells and are also found on TH17 cells, regulatory T cells, exhausted CD4^+^ T cells, as well as CD8^+^ T cells. TIM-3 expression is inversely correlated with IL-2 or IFN-γ production [16–18]. Following the interaction of TIM-3 with its ligands galectin-9 or CEACAM1, inhibitory signals are generated, leading to suppression of T cell activation [16, 19–21]. The prevalence of TIM-3 expressing T cells increases in various diseases such as chronic hepatitis C viral infection and cancers [22–25]. In vivo, TIM-3-blocking antibodies increase T cell immunity against chronic viral infections and cancers [22, 26–28]. Understanding how TIM-3 regulates T cell activation has been an important research focus on improving the protective immune response in chronic infections and cancers.

Given that blocking glucose or glutamine metabolism leads to T cell dysregulation and that TIM-3 negatively impacts T cell activation, it is possible that TIM-3 may downregulate glycolysis or glutaminolysis; however, to the best of our knowledge there are no studies that report the effect of TIM-3 on the T cell metabolism. In this study, we examined the effect of TIM-3 expression on glucose and glutamine metabolism as well as on mitochondrial content and function because mitochondria are involved in the glucose and glutamine metabolism. We found an association between TIM-3 expression and glucose consumption and lactate release but not with glutamine consumption, glutamate release, mitochondrial ROS production or mitochondrial membrane potential in Jurkat T cells. Also, we found that TIM-3 expression was linked to glucose uptake and GLUT1 expression levels.

## Results

### Glucose consumption and lactate release except glutamine consumption and glutamate release, decrease in Jurkat T cells according to TIM-3 overexpression

**Fig. 1:**
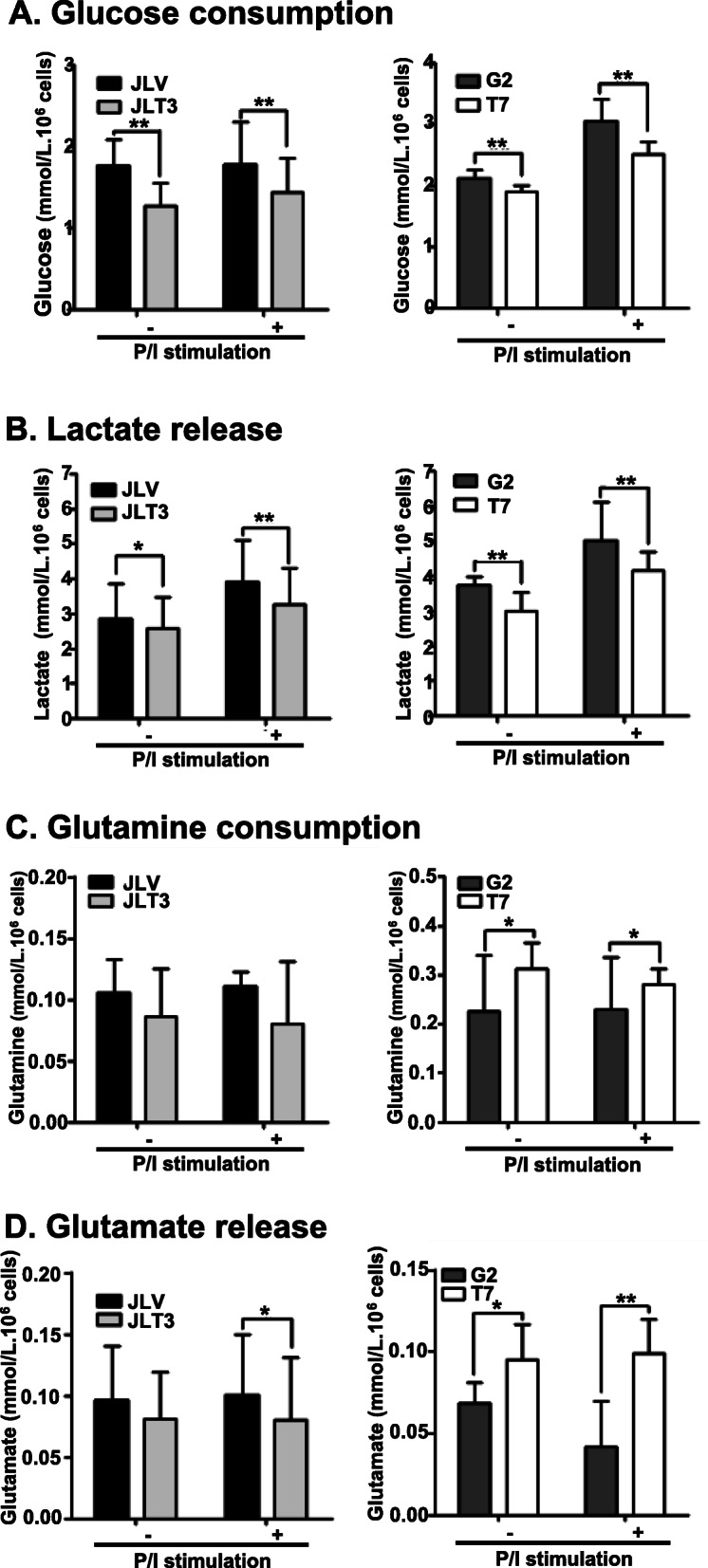
Glucose and glutamine metabolism in TIM-3 overexpressing cell lines. Glucose consumption (**a**), lactate release (**b**), glutamine consumption (**c**), and glutamate release (**d**), in control cells (JLV and G2) and TIM-3 overexpressing cells (JLT3 and T7) cultured in the absence (−) or presence (+) of PMA (25 ng/ml) and Iono (10 μM) for 6 h. A, B; Data of more than three independent experiments in quintuplicate or sextuplicate. C, D; Data represent two independent experiments performed in sextuplicate. Data are mean ± standard deviation (SD). P/I: PMA and Iono. *: *P* < 0.05

To examine whether TIM-3 expression was associated with glucose metabolism in T cells, we analyzed glucose consumption and the release of its metabolite, lactate, by two Jurkat T cell-derived cell lines that overexpress TIM-3; JLT3 expresses TIM-3 in the context of lentiviral vector and T7 expresses TIM-3 as well as GFP in the context of pIRES2-EGFP plasmid [29, 30]. JLT3 and T7 express TIM-3 in the absence of stimulation, and are significantly upregulated in the presence of PMA stimulation [30] (Suppl. Fig. 1). Glucose consumption and lactate release decreased significantly in the TIM-3 overexpressing cells compared to the control cells stably transfected with a corresponding empty vector both in the presence and absence of stimulation with PMA and ionomycin (Fig. 1a, b). We next analyzed glutamine metabolism in these cell lines. Glutamine consumption and glutamate release were similar between JLT3 and its control cell line, JLV, but increased significantly in T7 compared to its control cell line G2 (Fig. 1c, d). This discrepancy in glutamine consumption and glutamate release in JLT3 and T7 could be due to the differences in TIM-3 expression levels (Suppl. Fig. 1) or cellular properties attributed to the process of establishing stable cell lines such as the change in certain gene expression caused by the insertion of the vector into the host chromosomal DNA. If the latter is the case, increase in glutamine consumption and glutamate release in T7 could not be linked to TIM-3 expression. Taken together, these results are supportive of the association between TIM-3 expression and glucose metabolism but inconclusive for the association between TIM-3 expression and glutamine metabolism.

### Glucose consumption and lactate release increase in Jurkat T cells with TIM-3 knockout

**Fig. 2:**
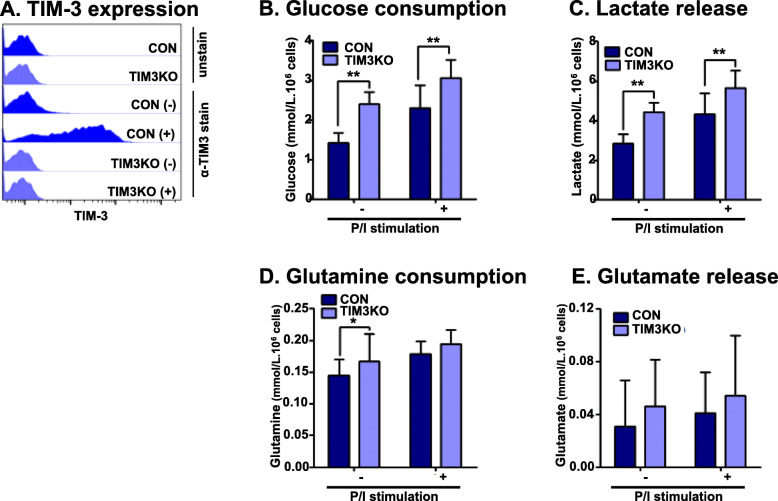
Glucose and glutamine metabolism in TIM-3 knockout Jurkat T cells**.** The surface expression of TIM-3 (**a**), glucose consumption (**b**), lactate release (**c**), glutamine consumption (**d**), and glutamate release (**e**) in control cells (CON) and TIM-3 knockout cells (TIM3KO) in the absence (−) or presence (+) of PMA (25 ng/ml) and Iono (10 μM) for 6 h. Data represent two or three independent experiments performed in sextuplicate. Data are mean ± SD. P/I: PMA and Iono. *: *P* < 0.05

To further examine the association of TIM-3 expression with glucose and glutamine metabolism, a TIM-3 knockout cell line was established (Fig. 2). Figure 2a shows no expression of TIM-3 in TIM3KO cells even after stimulation, while the basal and stimulation-induced TIM-3 expression was observed in the CON cells transfected with an empty vector. The association could be established if glucose consumption and lactate release increased but glutamine consumption and glutamate release decreased in the TIM-3 knockout cells. As expected, glucose consumption and lactate release increased significantly in TIM3KO cells compared to CON cells in the presence and absence of stimulation (Fig. 2b, c). However, glutamine consumption and glutamate release tended to be enhanced in TIM3KO cells compared to CON cells although statistical significance was observed only in glutamine consumption in the absence of stimulation (Fig. 2d, e). Considering the results of JLT3 and T7, these data were not supportive of the association between TIM-3 expression and glutamine consumption and glutamate release; however, these results strengthened the evidence for the involvement of TIM-3 expression in glucose metabolism of Jurkat T cells.

### TIM-3 cytoplasmic tail is required to decrease glucose consumption and lactate release in Jurkat T cells by TIM-3 overexpression

**Fig. 3:**
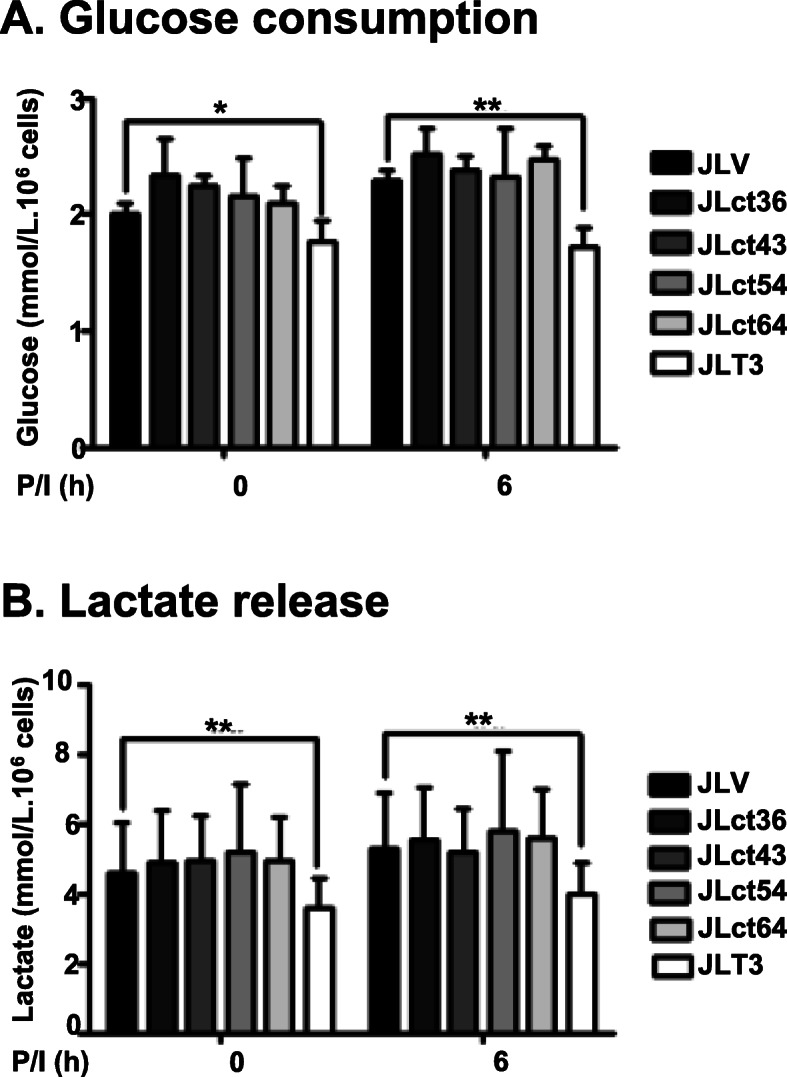
Glucose consumption and lactate release in cells expressing TIM-3 with deletion of cytoplasmic tail. Glucose consumption (**a**) and lactate release (**b**) in control cells (JLV), TIM-3 cytoplasmic tail deletional mutant cells (JLct36, JLct43, JLct54, and JLct64) and cells expressing full-length TIM-3 (JLT3) in the absence (−) or presence (+) of PMA (25 ng/ml) and Iono (10 μM) for 6 h. Data represent two independent experiments performed in sextuplicate. Data are mean ± SD. P/I: PMA and Iono. *: *P* < 0.05

We hypothesized that if TIM-3 was involved in glucose consumption and lactate release, TIM-3 signaling should be important and the cytoplasmic tail of TIM-3 (the signal-delivering part) was required for TIM-3-mediated effects on glucose consumption and lactate release. Therefore, glucose consumption and lactate release were assessed in JLct36, JLct43, JLct54, and JLct64 cell lines that express TIM-3 with a truncated cytoplasmic tail [30]. In contrast to JLT3 that expresses full-length TIM-3, these cell lines with truncated TIM-3 consumed and secreted similar amount of glucose and lactate, respectively, compared to JLV, the control cell line (Fig. 3a and b). These results indicate that the cytoplasmic tail of TIM-3 is critical to the regulation of glucose consumption and lactate release by TIM-3 in Jurkat T cells.

### TIM3 expression in Jurkat T cells is not associated with changes in mitochondrial mass, membrane potential, or ROS production

**Fig. 4:**
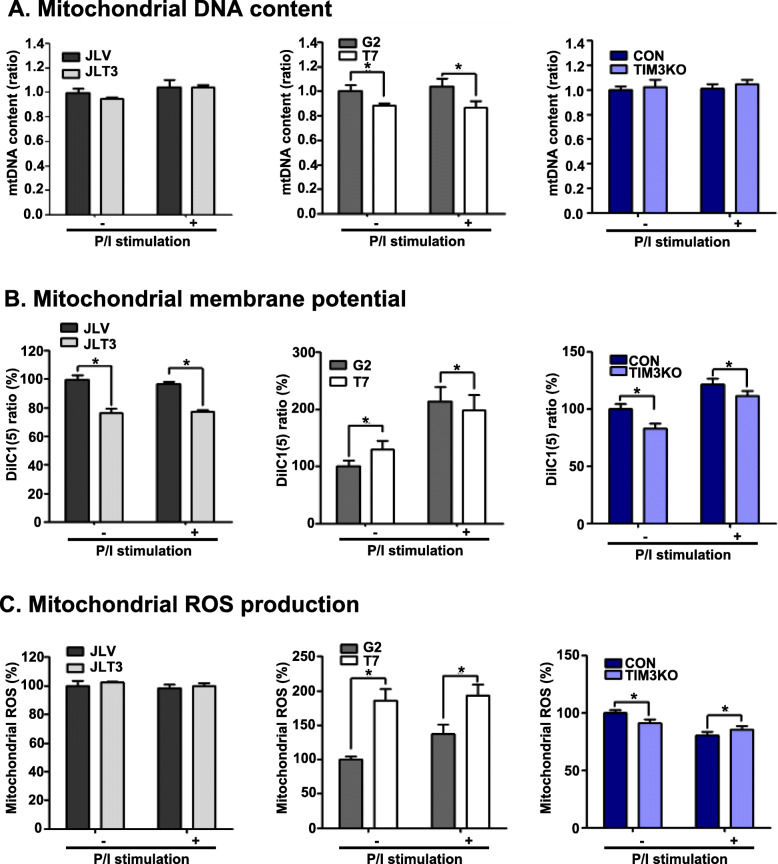
Mitochondrial DNA content, membrane potential, and ROS production in TIM-3 overexpressing or knockout cells. Mitochondrial DNA content (**a**), membrane potential (**b**), and ROS production (**c**), in control cells (JLV cell line, G2 clone, and CON clone), TIM-3 overexpressing cells (JLT3 cell line and T7 clone), and TIM-3 knockout cells (TIM3KO clone) cultured in the absence (−) or presence (+) of PMA (25 ng/ml) and Iono (10 μM) for 6 h. Mitochondrial DNA content was measured using qPCR. Mitochondrial membrane potential and ROS production were assessed by using flow cytometry with DilC(5) and MitoSOX™, respectively. Data represent two independent experiments performed in triplicates or sextuplets (JLT3 cell line and T7 clone). Data represent an experiment performed in sextuplets (TIM3KO clone). Data are mean ± SD. Mitochondrial DNA ratio: mitochondrial DNA to the nuclear 18S DNA. P/I: PMA and Iono. *: *P* < 0.05

Mitochondria contribute to cellular metabolism through the generation of ATP and regulation of the redox status [14]. To find out whether TIM-3 expression influences mitochondrial mass or function, we analyzed mitochondrial DNA content, membrane potential, and ROS production in JLT3, T7 and TIM3KO compared to their control cells. The reason was that parallel changes in mitochondrial mass or function of JLT3 and T7 and opposite changes in mitochondrial mass or function of TIM3KO compared to their control cell lines would support the possible association between TIM-3 and mitochondrial function. Mitochondrial DNA content was similar between JLT3 and JLV as well as between TIM3KO and CON but significantly less in T7 compared to G2 (Fig. 4a). Mitochondrial membrane potential decreased in JLT3 compared to JLV, whereas it increased in T7 compared to G2 in the absence of stimulation but decreased in the presence of stimulation. In the case of TIM3KO, the mitochondrial membrane potential decreased compared to that of CON cells regardless of stimulation (Fig. 4b). Mitochondrial ROS production was similar between JLT3 and JLV, but was significantly increased in T7 compared to G2; however, it slightly decreased in TIM3KO compared to CON in the absence of stimulation but not in the presence of stimulation (Fig. 4c). These results showed that TIM-3 expression might not be associated with the mitochondrial content, membrane potential, and ROS production in Jurkat T cells.

### TIM-3 expression decreases glucose uptake of Jurkat T cells at resting state and at an early time point of activation

**Fig. 5:**
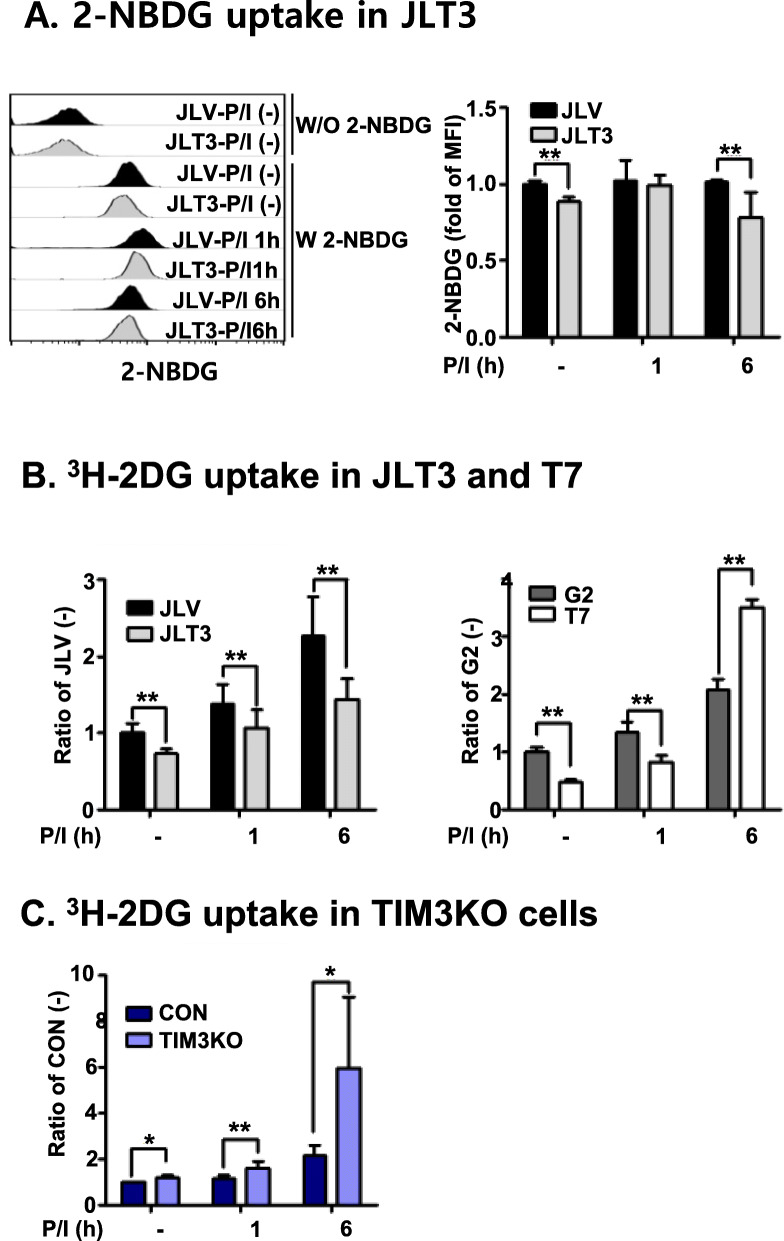
Glucose uptake by TIM-3 overexpressing or knockout cells. Glucose uptake by control cells (JLV, G2, and CON), TIM-3 overexpressing cells (JLT3 and T7), and TIM-3 knockout cells (TIM3KO) stimulated with PMA (25 ng/ml) and Iono (10 μM) for the indicated times was determined using a 2-NBDG (**a**), or ^3^H-2-DG uptake (**b** and **c**). Data represent two independent experiments performed in triplicate or sextuplicate. Data are mean ± SD. *: *P* < 0.005

We next examined how TIM-3 expression influenced glucose consumption and lactate release. Given that glucose consumption starts with glucose uptake, the association of TIM-3 expression with glucose uptake was investigated. The glucose uptake of JLT3 cells was measured using a 2-NBDG fluorescence-labeled glucose analog (Fig. 5a). The amount of 2-NBDG uptake by JLT3 was significantly less than that by control JLV before and after stimulation. Similar results were obtained when we assessed glucose uptake by JLT3 using ^3^H-labeled deoxy glucose (2DG, Fig. 5b). Also, glucose uptake by T7 cells was significantly reduced compared to the control G2 cells in the absence of stimulation or 1 h post-stimulation although it was significantly increased 6 h post-stimulation. Concomitantly, glucose uptake by TIM3KO was significantly higher than that by control cells in the absence and presence of stimulation (Fig. 5c). These results indicate that TIM-3 expression may be involved in modulating glucose uptake in Jurkat T cells.

### TIM-3 expression decreases Glut1 expression in Jurkat T cells at resting state and at an early time point of activation

**Fig. 6:**
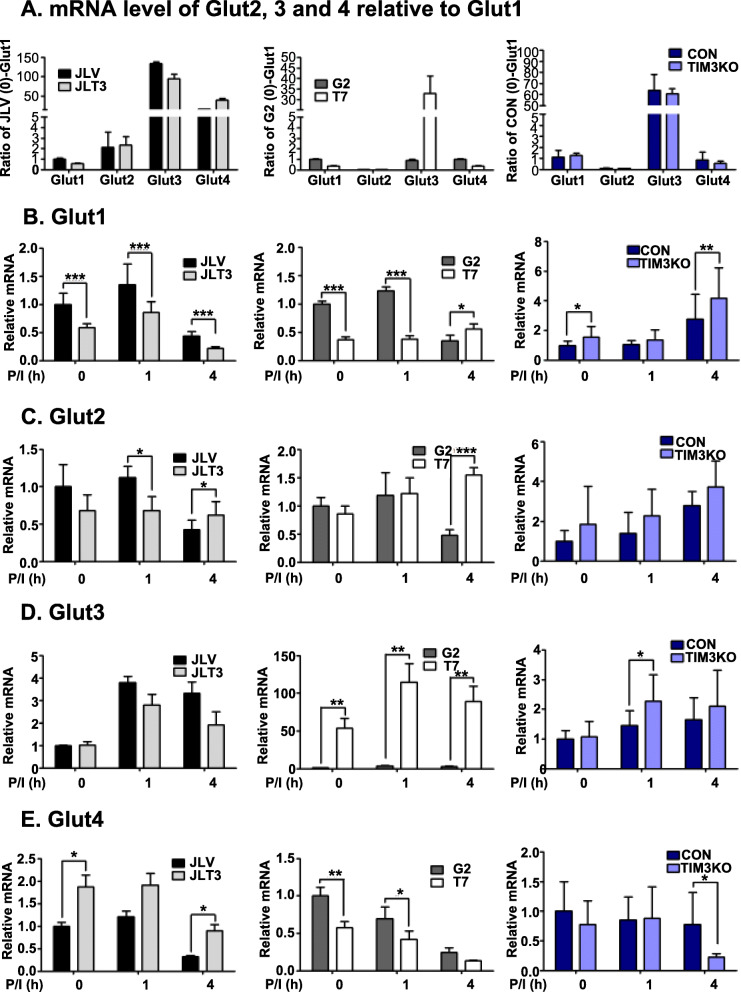
Transcript levels of Glut1, 2, 3 and 4 in TIM-3 overexpressing or knockout cells. Transcript levels of Glut2, 3, and 4 relative to Glut1 in each cell line in the absence of stimulation (**a**). mRNA levels of Glut1 (**b**), Glut2 (**c**), Glut3 (**d**), and Glut4 (**e**) in control cells (JLV, G2, and CON), TIM-3 overexpressing cells (JLT3 and T7), and TIM-3 knockout cells (TIM3KO) stimulated with PMA (25 ng/ml) and Iono (10 μM) for the indicated time was determined using qRT-PCR. Relative transcript: mRNA level of each cell line at the indicated time point relative to mRNA level in the corresponding control cells at the 0 time point. Data represent two independent experiments performed in triplicate or sextuplicate. Data are mean ± SD. *: *P* < 0.05

**Fig. 7:**
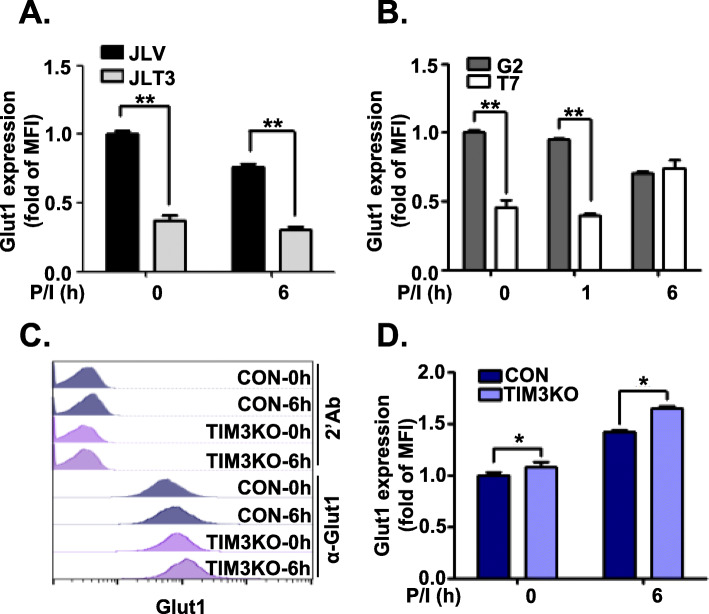
Glut1 protein level in TIM-3 overexpressing or knockout cells. Glut1 protein levels analyzed by using flow cytometry in TIM-3 overexpressing cells (**a** and **b**), and TIM-3 knockout cells (TIM3KO) (**c** and **d**) along with the corresponding control cells (JLV, G2, and CON, respectively). Cells stimulated for the indicated time with PMA (25 ng/ml) and Iono (10 μM) were labeled with PE-conjugated anti-mouse Ig Ab alone or together with mouse anti-Glut1 Ab and then the mean fluorescence intensity (MFI) was assessed. Representative histogram (**c**). Data represent two independent experiments performed in triplicate or sextuplicate. Data are mean ± SD. Fold of MFI: MFI relative to MFI of corresponding control cells at the 0 time point. P/I: PMA and Iono. *: *P* < 0.005

Human T cells take up glucose through glucose transporters such as Glut1 [31]. Therefore, it would be interesting to examine whether TIM-3 expression could affect the expression of Glut protein family members. We first examined the relative mRNA expression of *Glut1*, *Glut2, Glut3*, and *Glut4* in TIM3 overexpressing cell lines, TIM3KO cell line, and their respective control cell lines in the resting state by using quantitative RT-PCR (Fig. 6a). In all cell lines except the G2, *Glut3* transcripts were most abundant. In JLT3 and JLV cells, *Glut4* mRNA levels were higher than *Glut1* mRNA levels, but not in T7, G2, TIM3KO, and CON cells. We then examined the association between the transcript level of the Glut isotypes with TIM-3 expression before and after stimulation of the cells. *Glut1* transcript were less abundant in JLT3 and T7 than in JLV and G2, in the absence of stimulation and 1 h post-stimulation, whereas *Glut1* transcript were more abundant in TIM3KO than in CON (Fig. 6b). Thus, *Glut1* transcript level was associated with TIM-3 expression in the absence of stimulation and 1 h post-stimulation. In the case of *Glut2*, *Glut3,* and *Glut4* mRNAs, there was no correlation between their mRNA levels and TIM-3 expression (Fig. 6c, d, and e). These results indicate that TIM-3 expression may affect the expression of *Glut1* but not that of *Glut2, Glut3* or *Glut4*. Therefore, Glut1 protein expression was assessed in these cells (Fig. 7). Similar to *Glut1* mRNA levels, Glut1 protein expression was significantly lower in JLT3 than in JLV before and after stimulation (Fig. 7a). Also, Glut1 protein level was significantly lower in T7 than in G2 in the absence of stimulation and 1 h post-stimulation, but higher 6 h post-stimulation (Fig. 7b). In TIM3KO cells, Glut1 protein level was significantly increased compared to the CON cells both before and after stimulation (Fig. 7c and d). Taken together, these results suggest an association between TIM-3 and Glut1 expression, which may account for the link between TIM-3 expression and glucose uptake as well as glucose consumption.

## Discussion

TIM-3-mediated T cell regulation is a critical research agenda because manipulation of TIM-3 pathway may lead to enhancement of immunity against tumor cells or certain pathogens [22–27]. In this study, the possibility that TIM-3 may alter T cell activation-associated metabolism was evaluated, and three novel findings were demonstrated. Firstly, TIM-3 is involved in glucose metabolism but not glutamine consumption and release, mitochondrial DNA content and membrane potential, or ROS production. Secondly, there is a link between TIM-3 expression and glucose uptake as well as *Glut1* expression. Thirdly, the cytoplasmic tail of TIM-3 is required for its effect on glucose metabolism.

TIM-3 involvement in glucose consumption and lactate release was revealed using CD4^+^ Jurkat T cell-derived cell lines namely, two TIM-3 overexpressing JLT3 and T7, and a TIM3KO. Contrary to no apparent consistent results for glutamine consumption and glutamate release, mitochondrial DNA content and membrane potential, or ROS production, glucose consumption and lactate release were decreased in JLT3 and T7, but increased in TIM3KO, indicating that TIM-3 downregulates glucose consumption and lactate release. In line with our results, reduced glucose consumption was also reported in the exhausted T cells, of which approximately 30% expressed TIM-3, although 80% of these T cells also expressed other inhibitory molecules such as PD-1 and the role of TIM-3 in glucose consumption was not investigated [32]. The role of PD-1 in reduced glucose consumption was revealed in CD8^+^ T cells but not in CD4^+^ T cells [32]. Notably, PD-1 transcription was not detected in Jurkat T cells (data not shown). Thus, the novel finding of our study is the association between TIM-3 and glucose metabolism in CD4^+^ human T cell lines. Due to the limitation of Jurkat T cells that were derived from acute T cell leukemia, further study is needed to prove this finding by using primary T cells.

The decrease in glucose uptake and *Glut1* expression according to TIM-3 expression was demonstrated in Jurkat T cells. Glucose uptake and *Glut1* expression levels were lower in JLT3 and T7, but higher in TIM3KO. Thus, we propose that TIM-3 expression may affect glucose uptake and consumption partly through regulation of *Glut1* expression in a human T cell line. Glut1 is a principal glucose transporter required for the activation of human primary T cells which express Glut1, 3, 6, and 8 [31, 33]. The cell lines used in this study express Glut1, 2, 3, 4, 6, and 8 mRNAs and the relative amount of each mRNA vary between the cell lines (Fig. 6 and Suppl. Fig. 2). Our results show that Glut1 mRNA and protein levels, but not Glut2, 3, 4, 6 and 8 mRNA levels, are associated with TIM-3 expression (Fig. 6 and Suppl. Fig. 2A, B). Our results are similar to the previous report that Glut1 expression increased following T cell activation and decreased in exhausted T cells expressing PD-1 [32]. We ruled out the role of sodium-glucose linked transporter 1 (SGLT1), which are efficient at low extracellular glucose concentrations [34], in alteration of glucose uptake in a TIM-3 expression-dependent way by showing no association of SGLT1 transcript level with TIM-3 expression (Suppl. Fig. 2C). Thus, low Glut1 expression probably accounts for the reduced glucose uptake and consumption, and lactate release in TIM-3 expressing cells.

We should however mention that in TIM-3 expressing T7 cells, the reduced glucose uptake and Glut1 expression were transient, and increased 6 h post-stimulation compared to the control G2 cells. These finding suggest that additional mechanisms leading to reduced glucose consumption exist in the TIM-3 expressing cells. Given that hexokinase 2 (HK2) is the rate-limiting enzyme in glycolysis and that 6-phosphofructo-2-kinase/fructose-2,6-biphosphatase 3 (PFKFB3) directs glucose metabolism to either the pentose phosphate pathway or glycolysis-dependent pathway depending on its expression level and modification [35–38], we examined HK2 and PFKFB3 expression. However, any association between their expression and that of TIM-3 was not found (Suppl. Fig. 3 and  and Suppl. methods). Thus, the additional mechanisms by which TIM-3 reduces glucose consumption remain inconclusive.

Activation-induced glycolysis is required for cytokine production [8, 9], hence, TIM-3-associated reduction in glucose consumption and lactated release may contribute to decrease in the production of IL-2 and IFN-γ by TIM-3. Downregulation of IL-2 expression has been reported in JLT3 and T7 cells [30]. Therefore, we tested this hypothesis by assessing IL-2 production in JLT3 or T7 cells overexpressing Glut1. If the hypothesis is true, then IL-2 production should increase in these cells after Glut1 expression. Unfortunately, Glut1 overexpression was not achieved using the transfection of Glut1 expression vector into these cells. Further study is required to prove this hypothesis.

Since the cytoplasmic tail of TIM-3 mediates the delivery of signals and is essential for reduced glucose consumption and lactate release in TIM-3 overexpressing Jurkat T cells, expression of TIM-3 with a deletion in its cytoplasmic tail did not reduce glucose consumption and lactate release. It interacts with Bat3 or the long non-coding RNA, Lnc-Tim3, to activate distinct signaling pathways [39–41]. TIM-3 is also reported to interact with the interleukin-inducible T cell kinases (ITKs), Fyn and Lck, and p85 phosphatidylinositol 3-kinase (PI3K) and to regulate their activation [42]. PI3K/AKT/mTOR activation promotes Glut1 expression, glycolysis, and IL-2 production by T cells [32, 33, 36]. It is plausible that Glut1 expression and glucose consumption are modulated by signaling transmitted through the TIM-3 cytoplasmic tail in TIM-3 expressing Jurkat T cells.

## Conclusions

Our results show an association between TIM-3 expression and Jurkat T cell glucose metabolism. This association is a novel and significant finding in the alteration of T cell function by TIM-3 and raises the possibility of regulating TIM-3 expressing T cells through controlling glycolysis and/or glucose transporter expression. This is relevant to chronic infection and cancer in which increased frequency of hypofunctional TIM-3-expressing effector T cells have been observed.

## Methods

### Cell and culture

Cell lines over-expressing TIM-3 (JLT3 and JT7), cell lines expressing TIM-3 with a truncated cytoplasmic tail (JLct36, JLct43, JLct54, and JLct64), and control cells (JLV and JG2) were generated as described previously [29, 30]. Cells were grown in complete RPMI medium (Invitrogen, Life technologies, Gaithersburg, MD, USA) with 10% heat-inactivated FBS (Invitrogen), and penicillin/ streptomycin (Invitrogen). For activation, cells (1 × 10^6^ cells/well) were stimulated with phorbol 12-myristate 13-acetate (PMA; 25 ng/ml; Sigma-Aldrich, St Louis, MO, USA) and Ionomycin (Iono; 1 μM; Sigma-Aldrich) for an appropriate time.

### Establishment of TIM-3 knockout cell line

The pSpCas9(BB)-2A-GFP (PX458) plasmid (Addgene plasmid #48138) was used to generate TIM-3 knockouts [43]. Oligonucleotides for single guide RNA (sgRNA) for TIM-3 gene were cloned into the sgRNA scaffold of pSpCas9(BB)-2A-GFP (PX458) according to the Feng Zhang Lab CRISPR plasmid instructions [43]. The guide RNA sequences used for targeting TIM-3 gene were; 5′-CAC CGT TAT GCC TGG GAT TTG GAT CCG C-3′ and 5′-AAA CCC GGA TCC AAA TCC CAG GCA TAA C-3′. The construct was transfected into Jurkat T cells using the Neon Transfection System (Invitrogen). Transfected cells expressing green fluorescence were sorted 24 h post-transfection using the FACSAriaII (Becton–Dickinson, Franklin Lakes, NJ). The cells were further sorted according to the expression of TIM-3 after stimulation with PMA (25 ng) and Iono (1 μM) for 6 h by staining cells with a PE-conjugated anti-TIM-3 antibody (R&D Systems,Inc., Minneapolis, MN). After sorting four times, the cells were cloned by limiting dilution. The mutation in the TIM-3 genomic DNA sequence of the selected clone was confirmation by DNA sequencing.

### Quantitative PCR for glut transcription levels

Total RNA was isolated using RNAiso (TAKARA Bio Inc., Shiga, Japan) and subjected to reverse transcription using oligo (dT) primer and then real-time PCR using SYBR green Ex Taq Premix (TAKARA Bio, Inc.) with the appropriate primer sets and an ABI PRISM 7500 Sequence Detection System (Applied Biosystems, Foster, CA). The primers used were: Glucose transporter 1, Glut1-F; 5′-ACT GCA ACG GCT TAG ACT TCG AC-3′, Glut1-R; 5′-TCT CTG GGT AAC AGG GAT CAA ACA-3′, Glut2-F; 5′-TCC TGA GTG GCT TCT GA-3′, Glut2-R; 5′-GGC CAT AGA ATA GTT TGG CTA GAA T-3′, Glut3-F; 5′-CCT ATG CCG AAT GCC CTC A-3′, Glut3-R; 5′-TGA CAG TGC ACA TAC ATT CAAT CCT C-3′, Glut4-F; 5′-GGG CTG AGA CAG GGA CCA TAA C-3′, Glut4-R; 5′-CAT GAG CAA TGG CAT CCA GAA-3′, β-actin-F; 5′-TGG CAC CCA GCA CAA TGA A-3′, β-actin-R; 5′-CTA AGT CAT AGT CCG CCT AG-3′. All PCR results were normalized to β-actin mRNA.

### Assessment of Glut1 protein expression

The cells were fixed in 2% paraformaldehyde solution for 20 min at 24 °C, and subsequently washed with PBS. The fixed cells were permeabilized in PBS containing 0.3% saponin buffer for 15 min at room temperature. For blocking the Fc_γ_ receptors (FcRs), the cells were incubated with human FcR binding inhibitor (ebioscience, San Diego, CA) and then, incubated with anti-Glut1 antibody (Abcam, Cambridge, UK) for 1 h followed by PE- anti-mouse Ig antibody (ebioscience) for 1 h. Then, the cells were washed twice in PBS containing 0.1% saponin buffer, and resuspended in 0.25 mL 0.5% paraformaldehyde in PBS and analyzed using FACS CantoII (Becton–Dickinson).

### Assessment of glucose consumption, lactate release, glutamine consumption, and glutamate release

Glucose consumption, lactate release, glutamine consumption, and glutamate release were determined by measuring their concentrations in the final medium with a YSI biochemical analyzer (YSI Life Sciences; Simpsonville, SC). Cells (1 × 10^6^/ml) were either stimulated or not with PMA (25 ng) and Iono (1 μM) at 37 °C for 6 h and cell supernatant was harvested. Glucose or glutamine consumption and lactate or glutamate release were calculated as follows: consumption = mmol/L of ingredient in fresh complete RPMI media – mmol/L of ingredient in cultured media, release = mmol/L of ingredient in cultured media – mmol/L of ingredient in fresh complete RPMI. The rates were reported as mmol/L per 10^6^ cells.

### Assessment of glucose uptake

Glucose uptake was analyzed using the fluorescent glucose analog 2-(N-(7-nitrobenz-2-oxa-1, 3-diazol-4-yl) amino)-2-deoxyglucose (2-NBDG; Invitrogen) or Tritium (^3^H)-labeled 2-Deoxyglucose (2-DG; Perkin Elmer, Boston, MA). In the assessment using 2-NBDG, cells were unstimulated or stimulated with PMA (25 ng) and Iono (1 μM) at 37 °C for 1 h or 6 h, and then washed with N buffer (5 nM KCl, 2.5 mM CaCl_2_, 1 mM MgSO_4_, 1 mM KH_2_PO_4,_ 10 mM HEPES pH 7.2) to label with 2-NBDG by incubating them in N buffer containing 400 nM 2-NBDG and 140 mM NaCl at 37 °C for 10 min. Subsequently, cells were washed with Na^+^-free buffer twice and analyzed by FACS CantoII (Becton–Dickinson). In the assay using ^3^H-labeled 2-DG, unstimulated or stimulated cells as above were incubated in the presence of ^3^H-labeled 2-DG (5 μCi/mL) for 30 min. The uptake of ^3^H-labeled 2-DG was stopped by the addition of cold PBS containing the glucose transporter inhibitor cytochalasin B (2.5 μg/mL, Sigma-Aldrich). The cells were washed once with ice-cold PBS and lysed in 400 μL RIPA buffer with 1% SDS. Radioactive counts were determined using a scintillation counter.

### Assessment of mitochondrial function

We assessed mitochondrial membrane potential by labeling the cells with 25 nM 1,1′,3,3,3′,3′-hexamethylindodicarbo-cyanine iodide (DIlC(5), Invitrogen) for 30 min at 37 °C and then performed flow cytometry (Becton–Dickinson). Mitochondrial ROS was measured by flow cytometry following the labeling of cells with 5 μM MitoSOX™Red (Invitrogen) for 10 min at 37 °C prior to harvest.

### Assessment of mitochondrial DNA content

Cells (1 × 10^6^) were lysed with 500 μL of lysis buffer (100 mM NaCl, 10 mM Tris pH 8.0, 25 mM EDTA pH 8.0, 0.5% SDS, and 0.8 mg/ml protease K) and incubated at 56 °C overnight. Total DNA was isolated using the phenol/chloroform method and subjected to qPCR using primer sets specific to human mitochondrial DNA mt3212 to mt3319 (5′-CAC CCA AGA ACA GGG TTT GT-3′, human mt3319-R; 5′-TGG CCA TGG GTA TGT TGT TAA-3′) and nuclear 18S DNA (18S DNA-F; 5′-TAG AGG GAC AAG TGG CGT TC-3′, 18S DNA-R; 5′-CGC TGA GCC AGT CAG TGT-3′) [37]. A ratio of mitochondrial DNA to the nuclear 18S DNA was generated for each sample.

### Statistics

Student’s *t*-test was performed and *p*-values were determined to identify significant differences as indicated in the figures.

## Supplementary information

**Additional file 1.** Supplemental Figure 1. TIM-3 expression in TIM-3 overexpressing and control cells. Supplemental Figure 2. Transcript levels of Glut6, 8 and SGLT1 in TIM-3 overexpressing or knockout cells. Supplemental Figure 3. HK2 and PFKFB3 expression in TIM-3 overexpressing or knockout cells.

**Additional file 2.** Supplemental methods

## Data Availability

The datasets analysed during the current study are available from the corresponding author on reasonable request.

## Abbreviations

DilC(5): 1,1′,3,3,3′,3′-hexamethylindodicarbo-cyanine iodide
FcRs: Fcγ receptors
Glut: Glucose transporter
HK2: Hexokinase 2
Iono: Ionomycin
ITKs: Interleukin-inducible T cell kinases
mTOR: Mammalian target of rapamycin
PFKFB3: 6-phosphofructo-2-kinase/fructose-2,6-biphosphatase 3
PI3K: Phosphatidylinositol 3-kinase
PMA: Phorbol 12-myristate 13-acetate
ROS: Reactive oxygen species
SGLT1: Sodium-glucose linked transporter 1
TIM-3: T cell immunoglobulin and mucin domain containing protein-3
UDP-GlcNAc: Uridine diphosphate N-acetylglucosamine
2-NBDG: 2-(N-(7-nitrobenz-2-oxa-1, 3-diazol-4-yl) amino)-2-deoxyglucose
2-DG: 2-Deoxyglucose
