# Supplementary material for: Association of TIM-3 expression with glucose metabolism in Jurkat T cells
Source: BMC Immunol. 2020 Aug 20;21:48. doi: 10.1186/s12865-020-00377-6 (PMC7441550; doi:10.1186/s12865-020-00377-6)
Supplement: Supplementary file 1 — Additional file 1. Supplemental Figure 1. TIM-3 expression in TIM-3 overexpressing and control cells. Supplemental Figure 2. Transcript levels of Glut6, 8 and SGLT1 in TIM-3 overexpressing or knockout cells. Supplemental Figure 3. HK2 and PFKFB3 expression in TIM-3 overexpressing or knockout cells. [file 12865_2020_377_MOESM1_ESM.zip › Suppl fig legends edited.docx]

**Supplemental Figure 1. TIM-3 expression in TIM-3 overexpressing and control cells.** Surface expression of TIM-3 protein (A) in control cells (JLV cell line and G2 clone) and TIM-3 overexpressing cells (JLT3 cell line and T7 clone) stimulated with PMA (25 ng/ml) and Iono (10 μM) for 6 h was determined by using flow cytometry. Total TIM-3 protein level (B) was analyzed using western blotting. P/I: PMA and Iono.

**Supplemental Figure 2. Transcript levels of Glut6, 8 and SGLT1 in TIM-3 overexpressing or knockout cells.** Transcript levels of Glut6 (A), Glut8 (B) and SGLT1 (C) in control cells (JLV cell line, G2 clone, and CON clone), TIM-3 overexpressing cells (JLT3 cell line and T7 clone) and TIM-3 knockout cells (TIM3KO clone) stimulated with PMA (25 ng/ml) and Iono (10 μM) for 6 h was determined by using qRT-PCR. Relative transcript: mRNA level in each cell line at the indicated time point relative to mRNA level in the corresponding control cells at the 0 time point. Data represent experiments performed in triplicate. Data are mean ± SD. P/I: PMA and Iono. ND: not detected

**Supplemental Figure 3. HK2 and PFKFB3 expression in TIM-3 overexpressing or knockout cells.** The mRNA expression of HK2 (A) and PFKFB3 (B) in control cells (JLV cell line, G2 clone, and CON clone), TIM-3 overexpressing cells (JLT3 cell line and T7 clone), and TIM-3 knockout cells (TIM3KO clone) stimulated with PMA (25 ng/ml) and Iono (10 μM) for the indicated time was determined by using qRT-PCR. Relative transcript: mRNA level of each cell line at the indicated time point relative to mRNA level in the corresponding control cells at the 0 time point. PFKFB3 protein levels (C) were analyzed using western blotting. PFKFB3 protein band density was normalized to the actin band density. Data represent experiments performed in triplicate. Data are mean ± SD. P/I: PMA and Iono.
