# Supplementary material for: Association of TIM-3 expression with glucose metabolism in Jurkat T cells
Source: BMC Immunol. 2020 Aug 20;21:48. doi: 10.1186/s12865-020-00377-6 (PMC7441550; doi:10.1186/s12865-020-00377-6)
Supplement: Supplementary file 1 — Additional file 1. Supplemental Figure 1. TIM-3 expression in TIM-3 overexpressing and control cells. Supplemental Figure 2. Transcript levels of Glut6, 8 and SGLT1 in TIM-3 overexpressing or knockout cells. Supplemental Figure 3. HK2 and PFKFB3 expression in TIM-3 overexpressing or knockout cells. [file 12865_2020_377_MOESM1_ESM.zip › Supplemental Figures revised.pptx]

## Slide 1
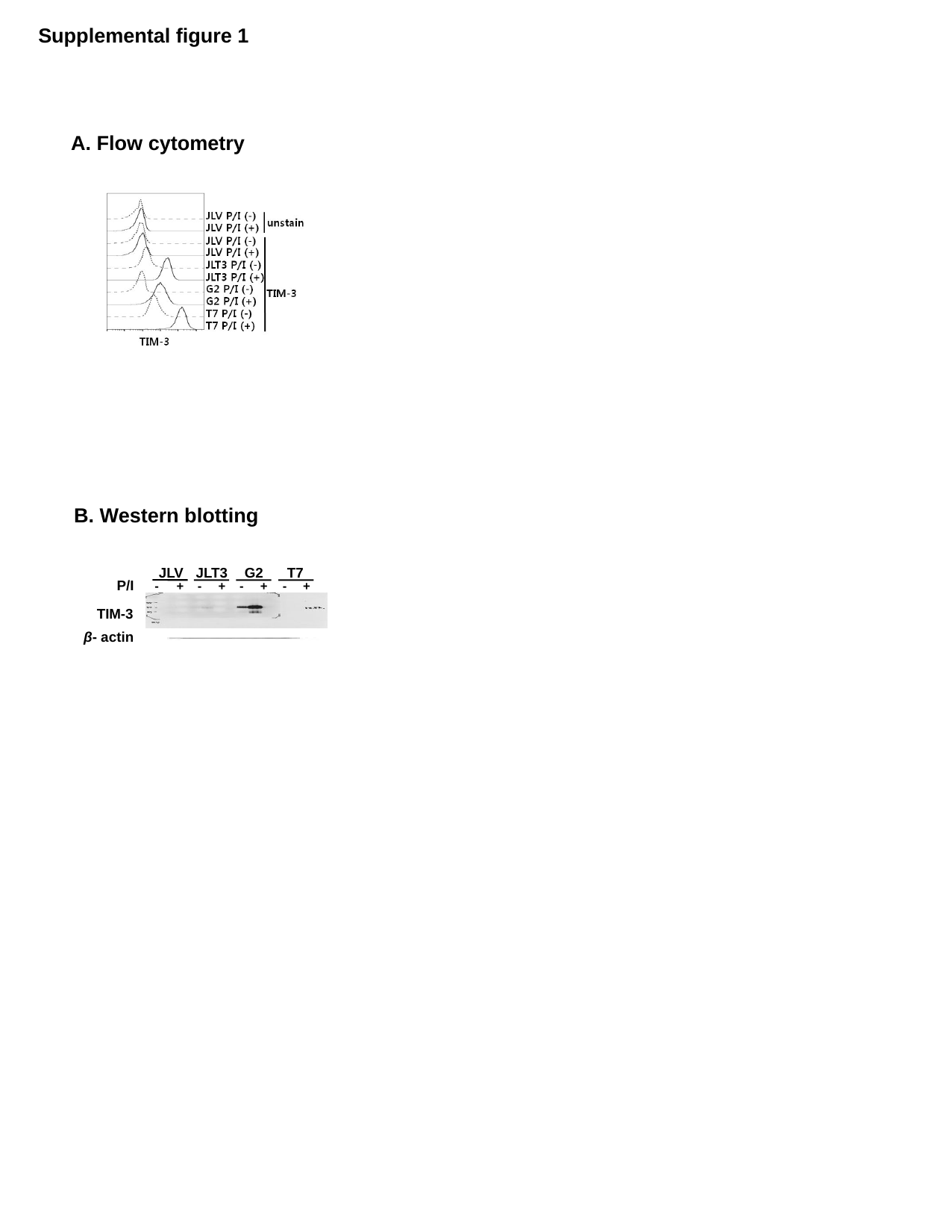

Supplemental figure 1
A. Flow cytometry
B. Western blotting
JLV
G2
T7
JLT3
P/I
+
-
+
-
+
-
+
-
TIM-3
β- actin

## Slide 2
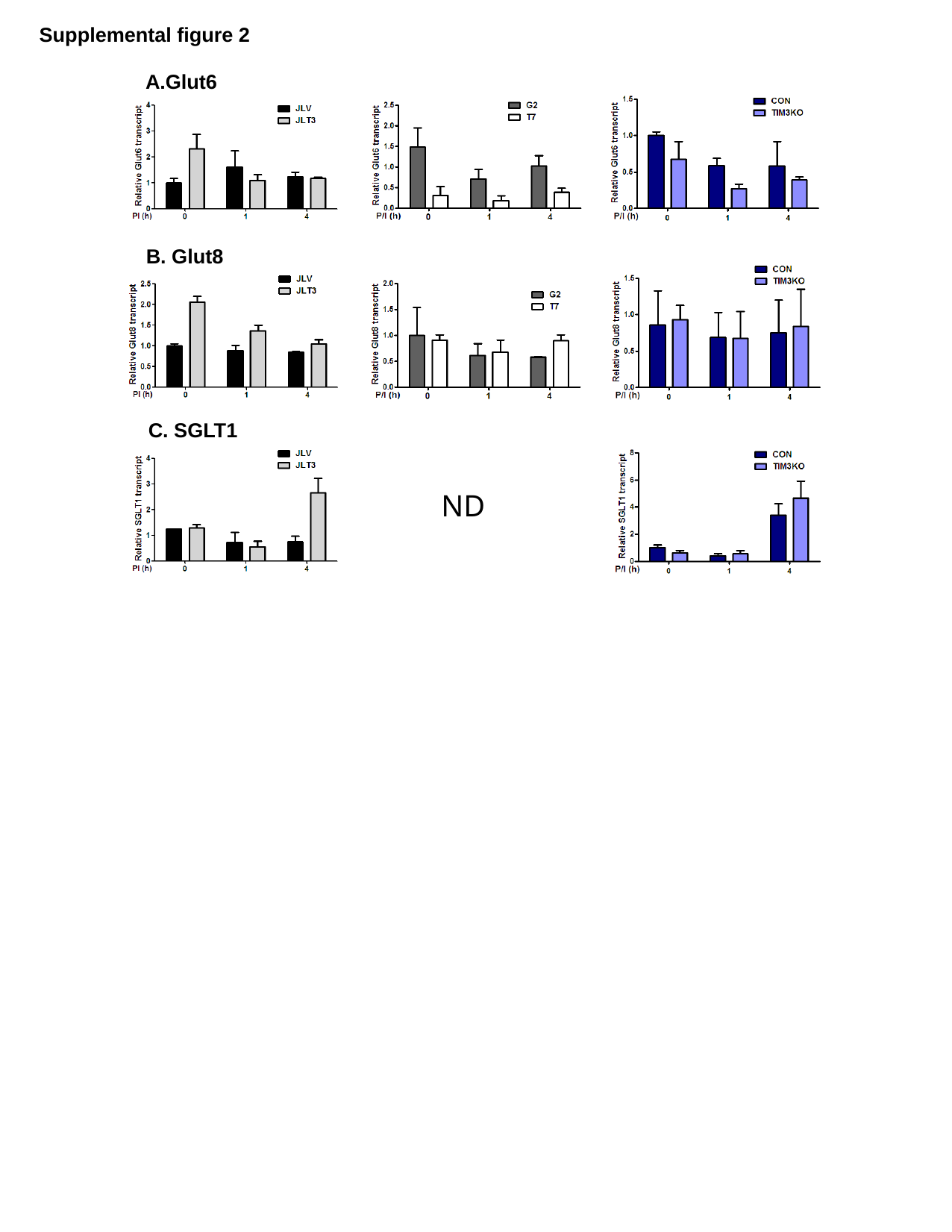

Supplemental figure 2
A.Glut6
B. Glut8
C. SGLT1
ND

## Slide 3
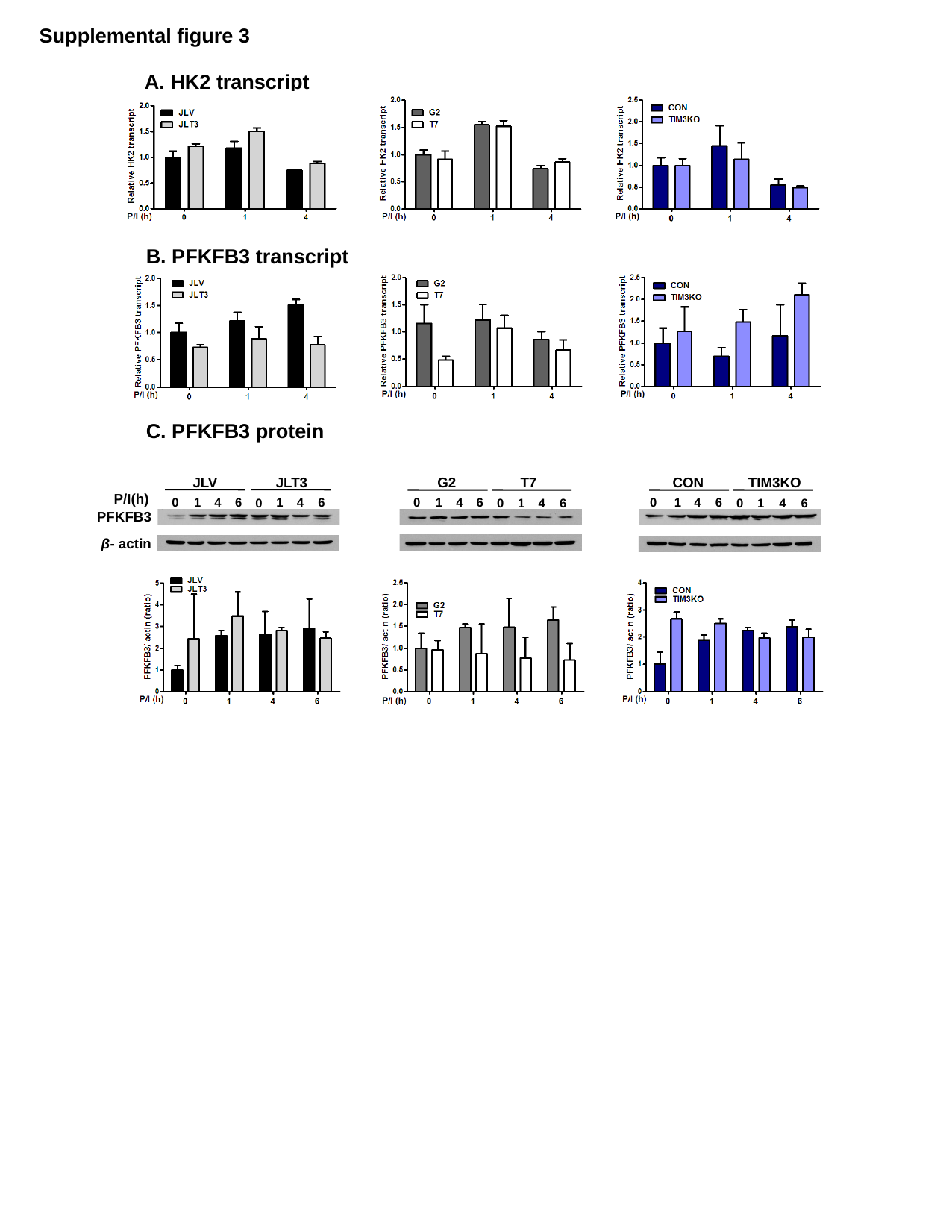

Supplemental figure 3
A. HK2 transcript
B. PFKFB3 transcript
C. PFKFB3 protein
T7
JLT3
G2
JLV
TIM3KO
CON
P/I(h)
1
4
6
0
1
4
6
1
4
6
0
0
1
4
6
0
1
4
6
1
4
6
0
0
PFKFB3
β- actin
