# Supplementary material for: Association of TIM-3 expression with glucose metabolism in Jurkat T cells
Source: BMC Immunol. 2020 Aug 20;21:48. doi: 10.1186/s12865-020-00377-6 (PMC7441550; doi:10.1186/s12865-020-00377-6)
Supplement: Supplementary file 2 — Additional file 2. Supplemental methods [file 12865_2020_377_MOESM2_ESM.docx]

**Supplemental methods**

**Quantitative PCR**

Total RNA was isolated using RNAiso (TAKARA Bio, Inc., Shiga, Japan) and subjected to real-time RT-PCR using SYBR green Ex Taq Premix (TAKARA Bio, Inc.) with the appropriate primer sets and an ABI PRISM 7500 Sequence Detection System (Applied Biosystems, Foster, CA). The primers used were: 6-phosphofructo-2-kinase/fructose-2,6-bisphosphatase 3 (PFKFB3)-F; 5’-CAT TTG GAA ACT CGG CAG AC-3’, PFKFB3-R; 5’-AGG ATC CAG GGC TCA CAG CA-3’, Hexokinase2 (HK2)-F; 5’-CAT AGA AAT ACC TCA TTC GCC TGT G-3’, HK2-R; 5’-AGC AAC CGC TTT GCC ATT C-3’, β-actin-F; 5’-TGG CAC CCA GCA CAA TGA A-3’, β-actin-R; 5’-CTA AGT CAT AGT CCG CCT AG-3’. All PCR results were normalized to β-actin mRNA.

**Western blotting**

Cells were lysed in RIPA lysis buffer (50 mM Tris-Cl pH 8.0, 150 mM NaCl, 0.5% SDS, 1% Triton X-100, 5% sodium deoxylcholate) containing protease inhibitors (Calbiochem, La Jolla, CA) and phosphatase inhibitors (Calbiochem) and then subjected to western blot analysis using anti-PFKFB3 antibody (Ab) (Abgent, Inc., San Diego, CA), anti-human TIM-3 Ab or anti-actin Ab (Bethyl laboratories, Inc., Montgomery, TX), as a primary Ab and using a horseradish peroxidase-conjugated anti-rabbit IgG Ab (Life Technologies, Carlsbad, CA) or anti-goat IgG Ab (Invitrogen) as a secondary Ab. Signals were detected using an enhanced chemical luminescence solution (Amersham, Piscataway, NJ). The PFKFB3 band intensity was normalized to the intensity of the actin band.
